# Assessing ChatGPT's Proficiency in Simplifying Radiological Reports for Healthcare Professionals and Patients

**DOI:** 10.7759/cureus.50881

**Published:** 2023-12-21

**Authors:** Pradosh Kumar Sarangi, Amrita Lumbani, M Sarthak Swarup, Suvankar Panda, Smruti Snigdha Sahoo, Pratisruti Hui, Anish Choudhary, Sudipta Mohakud, Ranjan Kumar Patel, Himel Mondal

**Affiliations:** 1 Radiodiagnosis, All India Institute of Medical Sciences, Deoghar, Deoghar, IND; 2 Physiology, Mayo Institute of Medical Sciences, Barabanki, IND; 3 Radiodiagnosis, Vardhman Mahavir Medical College and Safdarjung Hospital, New Delhi, IND; 4 Radiodiagnosis, SCB (Srirama Chandra Bhanja) Medical College and Hospital, Cuttack, IND; 5 Radiodiagnosis, All India Institute of Medical Sciences, Kalyani, Kalyani, IND; 6 Radiodiagnosis, Central Institute of Psychiatry, Ranchi, IND; 7 Radiodiagnosis, All India Institute of Medical Sciences, Bhubaneswar, Bhubaneswar, IND; 8 Physiology, All India Institute of Medical Sciences, Deoghar, Deoghar, IND

**Keywords:** artificial intelligence, large lqnguage model, hindi translation, health education, radiological report, patient-centered care, natural language processing, health literacy, healthcare communication, chatgpt

## Abstract

Background

Clear communication of radiological findings is crucial for effective healthcare decision-making. However, radiological reports are often complex with technical terminology, making them challenging for non-radiology healthcare professionals and patients to comprehend. Large language models like ChatGPT (Chat Generative Pre-trained Transformer, by OpenAI, San Francisco, CA) offer a potential solution by translating intricate reports into simplified language. This study aimed to assess the capability of ChatGPT-3.5 in simplifying radiological reports to facilitate improved understanding by healthcare professionals and patients.

Materials and methods

Nine radiological reports were taken for this study spanning various imaging modalities and medical conditions. These reports were used to ask ChatGPT a set of seven questions (describe the procedure, mention the key findings, express in a simple language, suggestions for further investigation, need of further investigation, grammatical or typing errors, and translation into Hindi). A total of eight radiologists rated the generated content in detailing, summarizing, simplifying content and language, factual correctness, further investigation, grammatical errors, and translation to Hindi.

Results

The highest score was obtained for detailing the report (94.17% accuracy) and the lowest score was for drawing conclusions for the patient (85% accuracy); case-wise scores were similar (p-value = 0.97). The Hindi translation by ChatGPT was not suitable for patient communication.

Conclusion

The current free version of ChatGPT-3.5 was able to simplify radiological reports effectively, removing technical jargon while preserving essential diagnostic information. The free version adeptly simplifies radiological reports, enhancing accessibility for healthcare professionals and patients. Hence, it has the potential to enhance medical communication, facilitating informed decision-making by healthcare professionals and patients.

## Introduction

ChatGPT, or Chat Generative Pre-trained Transformer, which is a state-of-the-art large language model (LLM) developed by OpenAI (San Francisco, CA), has demonstrated its prowess in understanding and generating human-like text across diverse domains [1]. The capability of ChatGPT extends to assisting both doctors and patients through its advanced natural language processing (NLP) abilities [2]. ChatGPT can serve as a valuable tool for doctors by offering quick access to medical literature, treatment guidelines, and diagnostic insights [3]. It can help doctors convey complex medical concepts in a more understandable manner to patients [4]. Patients can engage in informed discussions with ChatGPT and this can contribute to greater patient empowerment and understanding of their health journey.

In the realm of medical diagnostics and patient care, effective communication is paramount [5]. Radiological reports serve as a critical means of conveying complex medical information to both healthcare professionals and patients [6]. However, these reports often contain technical jargon and complex terminology that can pose challenges to comprehension among non-expert readers. In recent years, NLP models have shown remarkable advancements in various language-related tasks, raising the potential for their application in simplifying medical documents while preserving their clinical accuracy [7].

With this context, the objective of this study was to assess the extent to which ChatGPT can bridge the gap between technical radiological language and layman's terms, enhancing the comprehension and engagement of both medical practitioners and patients. By conducting a comprehensive analysis of the simplified reports produced by ChatGPT, we aimed to uncover the potential benefits and limitations of LLM in the field of radiology.

## Materials and methods

Study design

**Figure 1:**
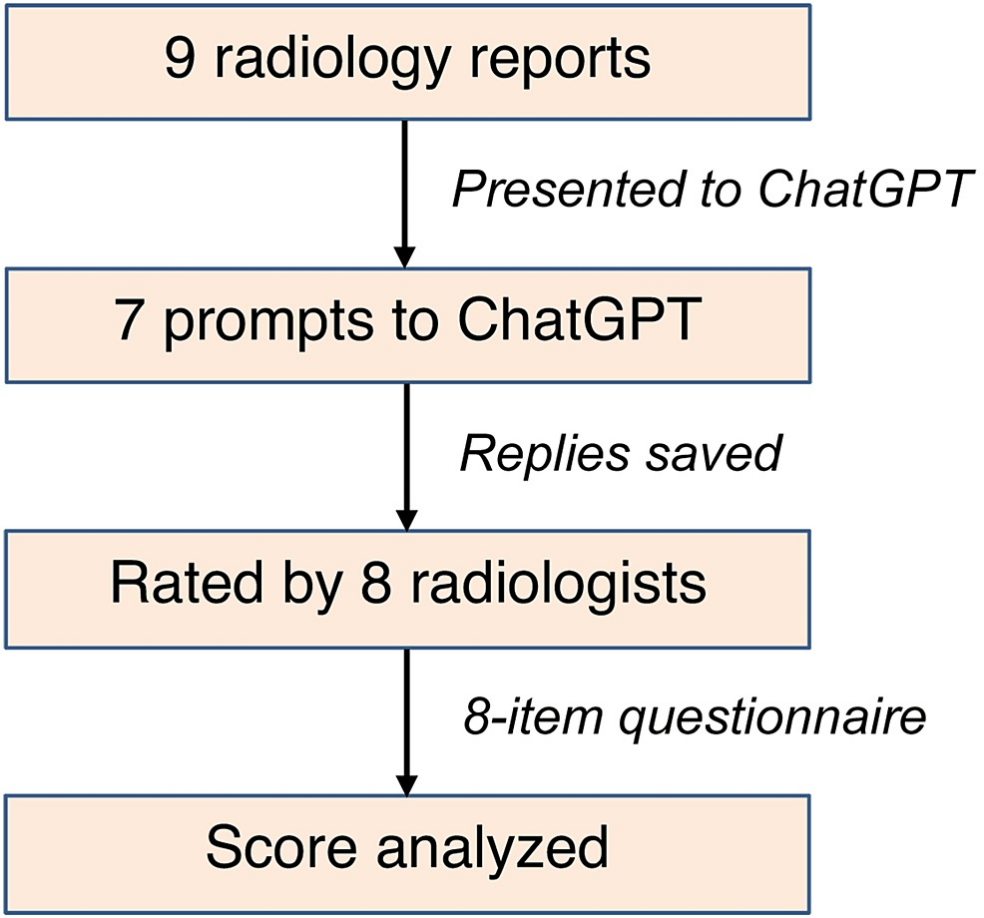
Brief steps of the study

This research employed a descriptive cross-sectional study design to assess the effectiveness of ChatGPT in simplifying radiological reports for diverse audiences, including healthcare professionals and patients. The brief procedure of the study is presented in Figure 1.

Radiological reports

A total of nine radiological reports were selected for this study. These reports encompassed a range of imaging modalities and medical conditions, ensuring a representative sample. Following are the cases, with impression/conclusion given in parentheses:

Case 1

Contrast-enhanced magnetic resonance imaging of the brain was performed (a known case of breast carcinoma with brain lesions favouring metastases).

Case 2

Contrast-enhanced computed tomography of the abdomen and pelvis was performed (a case of urinary bladder cancer, with transurethral resection of the bladder tumour showing necrotic retroperitoneal lymphadenopathy favouring metastases).

Case 3

Magnetic resonance imaging of the cervical spine and whole spine screening were performed (a case of diffuse marrow infiltrative lesions with multiple pathological collapse of vertebral bodies).

Case 4

High-resolution computed tomography of the thorax was performed (a case of COVID-19 pneumonia).

Case 5

Contrast-enhanced computed tomography of the abdomen and pelvis was performed (a case of uncomplicated acute interstitial pancreatitis).

Case 6

Magnetic resonance imaging of the left knee joint was performed (a case of complete anterior cruciate ligament tear, bucket handle tear of the medial meniscus and other associated findings).

Case 7

Non-contrast computed tomography of the brain was performed (normal study).

Case 8

Contrast-enhanced computed tomography of the neck was performed (a case of buccal carcinoma with necrotic cervical lymphadenopathy; radiological T stage, T4b; N stage, N2b).

Case 9

Non-contrast computed tomography of the left wrist joint was performed (a case of comminuted undisplaced fracture of the distal pole of the scaphoid without avascular necrosis).

**Figure 2:**
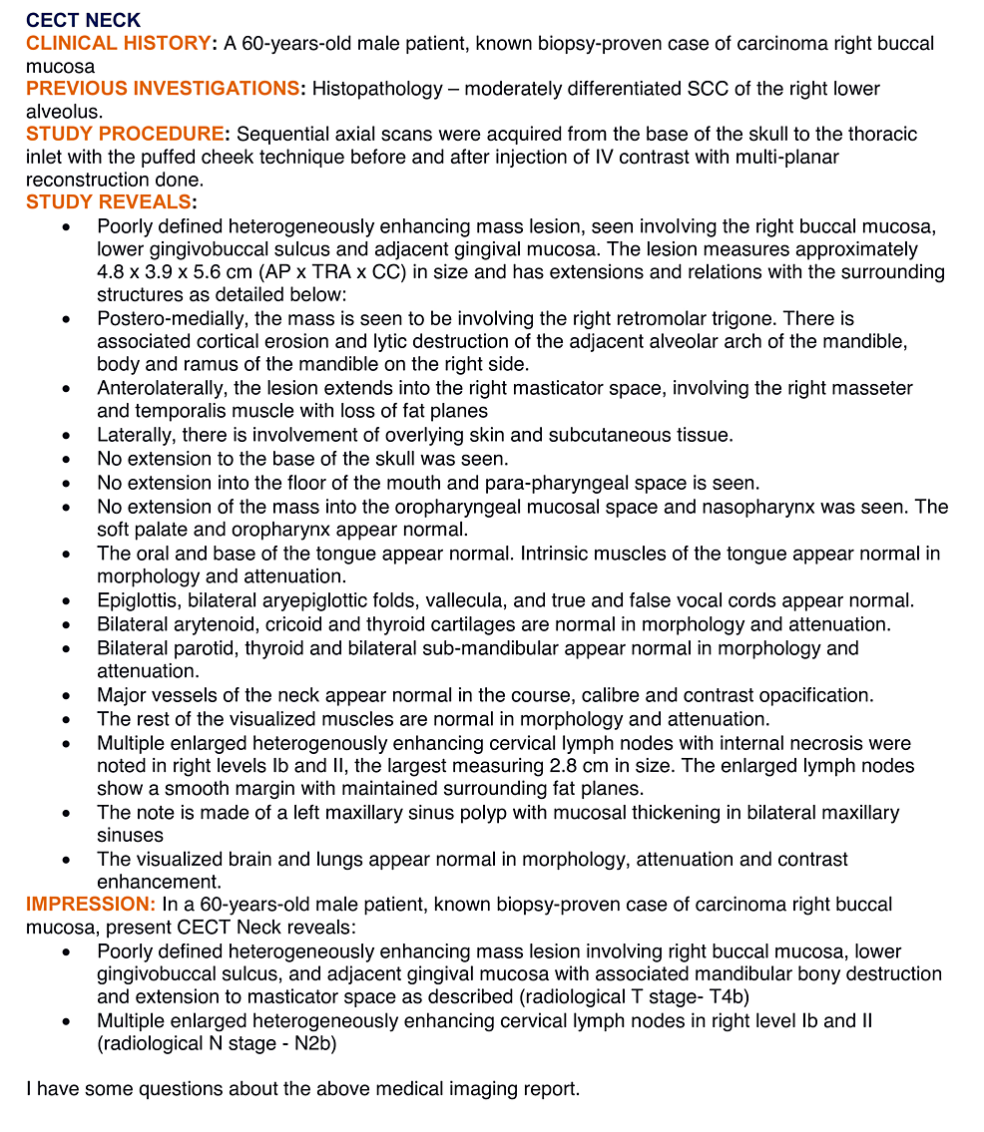
An example of a case used to generate response from ChatGPT CECT: contrast-enhanced computed tomography; SCC: squamous cell carcinoma; IV: intravenous; AP: anteroposterior; TRA: transverse; CC: craniocaudal

One such case is presented in Figure 2.

ChatGPT prompts

ChatGPT was asked an induction question along with the radiological report: “I have some questions pertaining to the above medical imaging report.” It replied, “Of course! I'd be happy to help answer any questions you have regarding the medical imaging report. Please feel free to ask your questions, and I'll do my best to provide you with the information you need.” In the following conversation, the prompts shown in Table 1 were asked.

**Table 1:** Prompts used to generate answers from ChatGPT

Number	Prompt
1	Describe the protocol or procedure technique in this study
2	Mention the key findings in the report in bullets
3	Explain this medical report in a simplified language
4	Does the report mention any further imaging or investigation?
5	Do I need any further imaging or investigation?
6	Are there any grammatical errors or typographical errors in the report?
7	Translate the report into the Hindi language

Rating items

An eight-question survey questionnaire was created to assess ChatGPT's ability to comprehend and elaborate on the provided reports. Questionnaire items encompassed multiple dimensions: the system's capacity to provide comprehensive details, its effectiveness in summarizing information, its aptitude for simplifying complex content and language, its accuracy in maintaining factual correctness, its potential to suggest further areas of investigation and its proficiency in identifying grammatical errors. In addition to those eight items, two items were used to assess the quality of translation into Hindi. Responses were measured on a five-point Likert-type scale, ranging from 'strongly disagree' (1) to 'strongly agree' (5). The questionnaire items are shown in Table 2.

**Table 2:** Questionnaire for the evaluation of the responses generated from ChatGPT *Reverse coding

Theme	Item	Response option				
		Strongly disagree	Disagree	Neither disagree nor agree	Agree	Strongly agree
Detail	The test protocol or procedure technique is described in detail	o	o	o	o	o
Key finding	Key findings are mentioned in the report	o	o	o	o	o
Simplicity (language)	The language of this report can be understood by a layperson	o	o	o	o	o
Simplicity (content)	The content of the report is easily understandable by a layperson	o	o	o	o	o
Factual correctness	The report is factually correct	o	o	o	o	o
Information	Relevant medical information for the patient is included in the report	o	o	o	o	o
Conclusion	The report leads patients to draw wrong conclusions*	o	o	o	o	o
Further suggestions	Suggestions about further imaging or investigation is justified	o	o	o	o	o
Additional components						
Hindi version completeness	The Hindi version of the report is complete	o	o	o	o	o
Grammar of the original report	There are grammatical errors in the original report*	o	o	o	o	o
Grammar of the Hindi version	There are grammatical errors in the Hindi version of the report*	o	o	o	o	o

Each radiologist was presented with the radiological reports in their original form and the versions simplified by ChatGPT. They were then tasked with evaluating the content using the developed questionnaire. Average ratings for each item were computed to determine the perceived effectiveness of ChatGPT. 

Calculation and data analysis

Descriptive statistics were employed to summarize the data, allowing for an understanding of ChatGPT's performance in different aspects of radiological report interpretation. Data were presented as means and standard deviations. As eight raters rated each case on an eight-item scale, each case had 64 scores (8 raters × 8 items) and each item had 72 scores (8 raters × 9 cases). The variance among scores was compared by one-way analysis of variance with post hoc test. A one-sample t-test was used to compare a variable with a hypothetical level and an unpaired t-test was used to compare two continuous variables. A p-value <0.05 was considered statistically significant.

Ethical considerations

This study involves the analysis of data generated from a language model that is freely available on the Internet. The cases used in this study were obtained from the departmental archive, which is used for teaching students, and these cases were fully anonymized. No human research participants were involved in this study. Therefore, this study did not require any ethical clearance.

## Results

**Figure 3:**
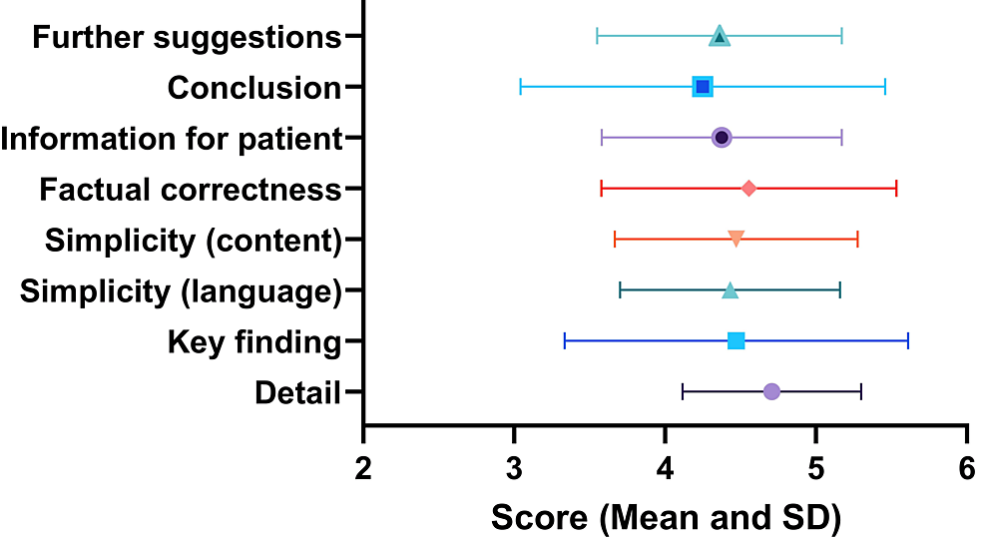
Item-wise scores of the ChatGPT-generated content during the interpretation of radiology reports

A total of eight raters rated nine cases with an eight-item questionnaire; all of the responses were complete and taken for final analysis. Themes and relevant average scores are shown in Figure 3.

The highest score was obtained for detailing of the report (4.71±0.59) and the lowest score was for drawing conclusion for the patient (4.25±1.21). However, theme-wise scores were similar for all the themes and there was no statistically significant difference (p-value = 0.88).

**Figure 4:**
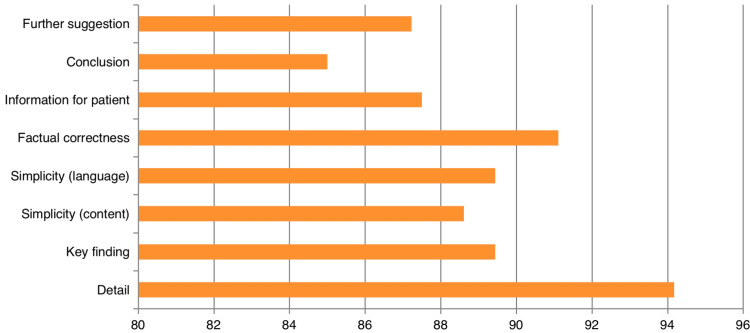
Percentages of scores of the ChatGPT-generated content during the interpretation of radiology reports

Item-wise percentages of the scores showed a range of 85% to 94.17% as shown in Figure 4.

**Figure 5:**
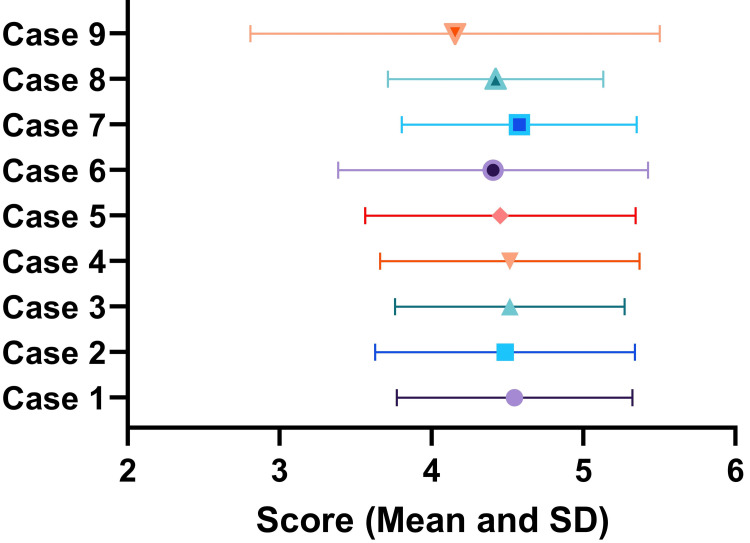
Case-wise scores of the ChatGPT-generated content during the interpretation of radiology reports

Case-wise average scores are shown in Figure 5. Case-wise scores were also not significantly different from each other (p-value = 0.97). Average scores ranged from 4.57±0.77 for case 7 to 4.16±1.34 for case 9.

**Figure 6:**
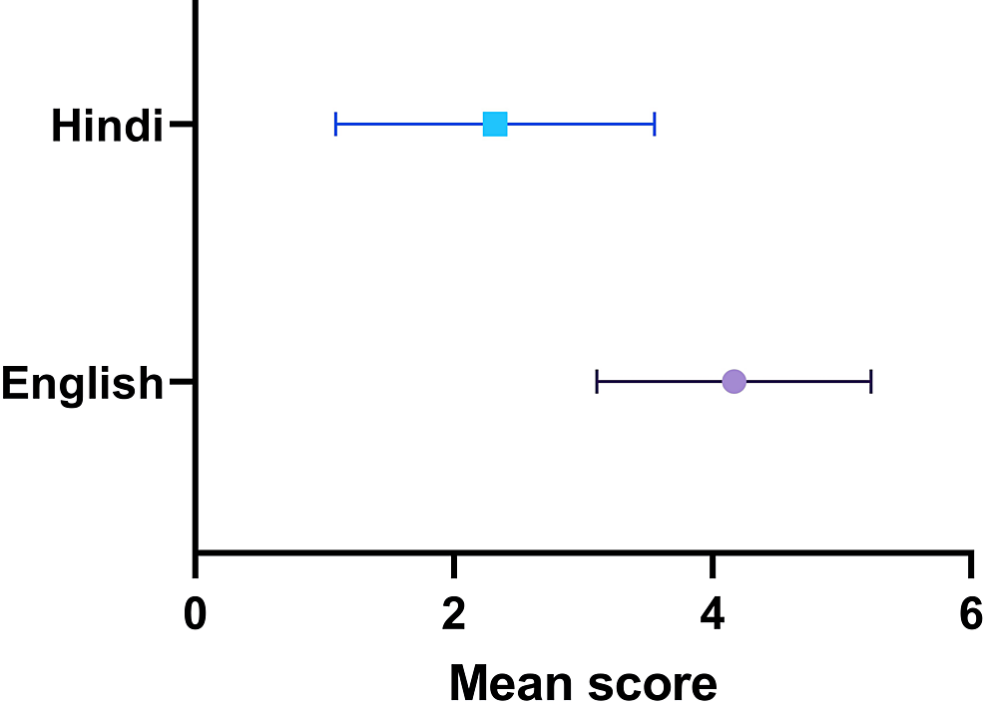
Average scores for grammatical correctness of the original report and the Hindi version of the report

The Hindi translation by ChatGPT was not suitable for patient communication. We took 80% (a hypothetical score of 4) accuracy as the minimum criterion. The completeness score was 2.89±1.51 that was significantly lower than the minimum score of 4 (p<0.0001, 95% CI of discrepancy -1.465 to -0.757). The grammatical correctness score further decreased to a mean score of 2.32±1.23 (p<0.0001, 95% CI of discrepancy -1.970 to -1.391) that was also significantly lower when compared to the grammatical characteristics of the original report (t-test p<0.0001) (Figure 6).

There was a good level of agreement among the raters (intraclass correlation coefficient for the average measure was 0.873; 95% CI 0.829-0.909; p<0.0001). Correlations among the scores by eight raters are shown in Table 3.

**Table 3:** Correlation matrix of scores obtained for nine cases on 11 items

Rater	Rater 1	Rater 2	Rater 3	Rater 4	Rater 5	Rater 6	Rater 7	Rater 8
Rater 1	1							
Rater 2	.605	1						
Rater 3	.367	.446	1					
Rater 4	.226	.324	.325	1				
Rater 5	.607	.517	.258	.011	1			
Rater 6	.623	.632	.473	.343	.523	1		
Rater 7	.621	.932	.559	.343	.506	.641	1	
Rater 8	.736	.518	.282	.259	.589	.577	.586	1

## Discussion

We found that ChatGPT achieves a high level of accuracy in interpreting and generating content for radiological reports. The possible reason for this finding may be attributed to its comprehensive training dataset, advanced transformer architecture, and language understanding capabilities. Its exposure to diverse medical literature and technical documents might have enabled it to grasp the intricacies of radiology terminology and concepts [8]. The transformer architecture empowers ChatGPT to capture contextual relationships effectively, allowing it to generate coherent descriptions of radiological findings [9]. Hence, ChatGPT may help in making a radiology report more suitable for understanding for a patient or non-radiologist healthcare professionals [10].

A study by Bhayana et al. found that despite no specialized training in radiology, ChatGPT nearly passed a radiology board-style examination without images [11]. Furthermore, Rao et al. found that using ChatGPT for radiologic decision-making is feasible, with the potential to enhance clinical workflow and the responsible use of radiology resources [12]. Hence, LLMs like ChatGPT-3.5 have the potential to enhance radiology reporting and patient engagement by automating the development of a radiologist report's clinical history and impression, producing layman reports, and offering patients essential questions and answers concerning radiology report results [13]. Our study supports the above notion and found that ChatGPT can effectively interpret a radiology report and provide further information regarding the report.

In contrast, Currie et al. reported that in radiology examination, ChatGPT lacked the depth and breadth of knowledge and scored below the level of an average medical student [14]. In support, Wagner et al. found that only two-thirds of the questions from radiologists' regular clinical practice were answered correctly by ChatGPT-3, with the other replies including mistakes. The bulk of the references offered were not located, and just a minority of the references provided included the correct information to answer the question [15]. Hence, both positive and negative results are being generated by artificial intelligence. Patil et al. concluded that although chatbots have adequate radiological expertise, they may generate erroneous or illogical answer explanations and do not always address the question's instructional substance [16]. Lecler et al. also supported this view and concluded that ChatGPT answers numerous questions that radiologists may have about ChatGPT and outlines the possible benefits ChatGPT may bring in their everyday practice as well as present limitations [17]. Hence, further exploration and improvements are warranted for the full-fledged use of LLM in radiological report interpretation [18].

The consistent generation of reports by ChatGPT across all cases can be attributed to its deterministic nature and the uniformity of the input prompts. ChatGPT operates based on patterns it has learned from its training data, and when presented with similar or identical prompts, it tends to produce similar responses. As the prompts were structured in a comparable manner, the model's responses were likely to reflect this consistency [19]. Variations in input phrasing may generate inappropriate content. However, this would be a future research topic.

The finding that the Hindi translations produced by ChatGPT were unsuitable for effective patient communication can be attributed to several key factors. The translations did not meet the minimum accuracy criteria of 80%, indicating a lack of precision in conveying the intended information. Similarly, Google Translate (Google, Mountain View, CA) was also not impressive in the translation of survey questionnaires [20]. Translated content failed to capture the entirety of the original material. Additionally, the decline in grammatical correctness further compounds the issue, with the translated content deviating significantly from the original report's grammatical characteristics. This deficiency in maintaining linguistic and contextual accuracy highlights the challenges in producing suitable translations for effective patient communication. Hence, at this point, ChatGPT may not be suitable for Hindi translation for communicating radiological reports.

The observed strong agreement among the raters can be attributed to several contributing factors. The utilization of well-defined assessment criteria and clear guidelines provided to the raters might play a pivotal role in reducing ambiguity and subjectivity. Moreover, possible uniform training might enhance the raters' consistent rating. The relatively limited number of cases evaluated might have facilitated agreement due to focused attention and reduced variability.

Limitations

One notable limitation of this study is its relatively small number of reports that impacted the generalizability of the findings. In addition, reliance on a specific questionnaire for assessment might introduce biases or overlook nuanced aspects of the evaluated content. Moreover, the study's focus on specific themes and dimensions of content evaluation might have led to the neglect of other important criteria that could influence the overall quality assessment. Also, the study design does not account for potential changes in the model's performance over time or its sensitivity to different input phrasings. While efforts were made to ensure raters’ quantitative ratings, subjectivity in assessments might still exist due to individual interpretation differences.

## Conclusions

This study provides valuable insights into the content generated by ChatGPT by interpreting a radiology report. While the model excels in certain aspects, such as detailing the report, it faces challenges in translating to Hindi. The present free version of ChatGPT-3.5 may be a valuable tool for simplifying radiological reports for non-radiologist healthcare professionals and patients, helping them make informed clinical decisions or get answers related to the report. The model's ability to bridge the gap between specialized terminology and accessible communication holds great promise for enhancing interdisciplinary collaboration and improving patient care across diverse medical contexts. Despite the limitations, the study contributes to our understanding of ChatGPT's capabilities and provides a foundation for future advancements in content generation and evaluation.

## References

[1] Lubowitz JH (2023). ChatGPT, an artificial intelligence chatbot, is impacting medical literature. Arthroscopy.

[2] Dave T, Athaluri SA, Singh S (2023). ChatGPT in medicine: an overview of its applications, advantages, limitations, future prospects, and ethical considerations. Front Artif Intell.

[3] Ruksakulpiwat S, Kumar A, Ajibade A (2023). Using ChatGPT in medical research: current status and future directions. J Multidiscip Healthc.

[4] Mondal H, Mondal S, Podder I (2023). Using ChatGPT for writing articles for patients' education for dermatological diseases: a pilot study. Indian Dermatol Online J.

[5] Ifrim RA, Klugarová J, Măguriță D, Zazu M, Mazilu DC, Klugar M (2022). Communication, an important link between healthcare providers: a best practice implementation project. JBI Evid Implement.

[6] Fatahi N, Krupic F, Hellström M (2019). Difficulties and possibilities in communication between referring clinicians and radiologists: perspective of clinicians. J Multidiscip Healthc.

[7] Casey A, Davidson E, Poon M (2021). A systematic review of natural language processing applied to radiology reports. BMC Med Inform Decis Mak.

[8] Liu J, Wang C, Liu S (2023). Utility of ChatGPT in clinical practice. J Med Internet Res.

[9] Srivastav S, Chandrakar R, Gupta S (2023). ChatGPT in radiology: the advantages and limitations of artificial intelligence for medical imaging diagnosis. Cureus.

[10] Şendur HN, Şendur AB, Cerit MN (2023). ChatGPT from radiologists' perspective. Br J Radiol.

[11] Bhayana R, Krishna S, Bleakney RR (2023). Performance of ChatGPT on a radiology board-style examination: insights into current strengths and limitations. Radiology.

[12] Rao A, Kim J, Kamineni M, Pang M, Lie W, Dreyer KJ, Succi MD (2023). Evaluating GPT as an adjunct for radiologic decision making: GPT-4 versus GPT-3.5 in a breast imaging pilot. J Am Coll Radiol.

[13] Elkassem AA, Smith AD (2023). Potential use cases for ChatGPT in radiology reporting. AJR Am J Roentgenol.

[14] Currie G, Singh C, Nelson T, Nabasenja C, Al-Hayek Y, Spuur K (2023). ChatGPT in medical imaging higher education. Radiography (Lond).

[15] Wagner MW, Ertl-Wagner BB (2023). Accuracy of information and references using ChatGPT-3 for retrieval of clinical radiological information. Can Assoc Radiol J.

[16] Patil NS, Huang RS, van der Pol CB, Larocque N (2023). Comparative performance of ChatGPT and Bard in a text-based radiology knowledge assessment. Can Assoc Radiol J.

[17] Lecler A, Duron L, Soyer P (2023). Revolutionizing radiology with GPT-based models: current applications, future possibilities and limitations of ChatGPT. Diagn Interv Imaging.

[18] Ray PP, Majumder P (2023). ChatGPT in radiology: a deeper look into its limitations and potential pathways for improvement. Can Assoc Radiol J.

[19] Nguyen J, Pepping CA (2023). The application of ChatGPT in healthcare progress notes: a commentary from a clinical and research perspective. Clin Transl Med.

[20] Mondal H, Mondal S, Mondal S (2019). Feasibility of using “Google Translate” in adaptation of survey questionnaire from English to Bengali: a pilot study. Indian J Soc Psychiatry.

